# Cardiac surgical procedures for the coronary sequelae of Kawasaki disease

**DOI:** 10.3402/ljm.v7i0.19796

**Published:** 2012-12-03

**Authors:** Shi-Min Yuan

**Affiliations:** Department of Cardiothoracic Surgery, The First Hospital of Putian, Teaching Hospital, Fujian Medical University, Putian, Fujian Province, People's Republic of China

**Keywords:** cardiac surgical procedures, coronary aneurysm, coronary artery bypass, coronary stenosis, mucocutaneous lymph node syndrome

## Abstract

**Objectives:**

The aim of this article is to make an evaluation on the clinical features of patients with Kawasaki disease who require a cardiac surgical procedure including coronary artery bypass grafting, coronary arterial aneurysmorrhaphy or heart transplantation.

**Methods:**

English literature of Kawasaki disease for cardiac surgery (1990–2011) was retrieved in the Pubmed database. The clinical features of the patient setting from the representative articles were collected and analyzed.

**Results:**

Patients with Kawasaki disease were very young, with some requiring a cardiac surgical procedure at a very early age. The interval between the onset and the surgical operation was 9.5±9.4 years. The prevalence of myocardial infarction and re-infarction was high. Giant aneurysm, critical stenosis with calcification and thrombus formation of the coronary arteries often warrant coronary artery bypass, heart transplantation or coronary arterial aneurysm plication. The left internal mammary artery to the left anterior descending coronary artery was the most commonly used graft in coronary artery bypass. Graft patency rate was 82.4% at 21.4±32.3 (range 0.1–252) month follow-up. The early and late mortalities of this patient setting were 0.6 and 3.0%, respectively.

**Conclusions:**

Patients with Kawasaki disease may develop coronary artery lesions prone to aneurysmal formation with calcification and thrombus and may require coronary artery bypass at a very early age. With the left internal mammary artery as the first choice of bypass graft, the long-term patency and patient survival was satisfactory.

Kawasaki disease, also known as mucocutaneous lymph node syndrome, is an autoimmune disease affecting the blood vessels of the whole body, characterized by the typical changes in the mucous membranes and the enlarged lymph nodes. Poor response to antibiotics is a feature of the disease (1). It occurs worldwide, most prevalent in Japan and East Asian countries: 218.6 per 100,000 children from 0 to 4 years of age in the year 2008, 69 per 100,000 children under 5 years in Taiwan, 86.4 per 100,000 in Korea, 20.8 per 100,000 in the USA, and 8.39 per 100,000 in England (2). In Finland, the annual prevalence of Kawasaki disease was 3.1–7.2 per 100,000 children younger than 5 years of age (3). Eighteen cases of Kawasaki disease were reported in Scotland (4). However, it has been described that susceptibility to Kawasaki disease depends upon racial factors more than geographic reasons (5).

One of the predominant sequelae of Kawasaki disease is coronary artery disease. Among children with Kawasaki disease, 15–25% may develop coronary aneurysms (6). According to Kato's report, 55% of the small or moderate-sized aneurysms were fully regressed within 2 years of follow-up, and 4.7% of the patients might develop myocardial infarction (7). Even though Kawasaki disease has been continuously reported, the clinical features of Kawasaki disease in terms of cardiovascular surgical aspects were not sufficiently discussed. The aim of this article is to make an evaluation of the clinical features of patients with Kawasaki disease requiring a cardiac surgical procedure.

## Methods

English literature of Kawasaki disease patients who required a cardiac surgical procedure including coronary artery bypass grafting (CABG), coronary arterial aneurysmorrhaphy or heart transplantation (1990–2011) was retrieved in the Pubmed database. The clinical features of the patient setting from the representative articles were collected and analyzed. Data were expressed as mean ±s tandard deviation. Unpaired *t*-test was used to compare the quantitative data when necessary and *p*<0.05 was considered to be of statistical significance.

## Results

A total of 71 representative articles (nine original articles/larger series and 62 case reports or cases series) (8–78) were collected. These articles involved 637 patients with Kawasaki disease who were undergoing a cardiac surgical procedure.

Of the patients whose gender was given, 88 were males and 28 were females with a male-to-female ratio of 3.1:1. Age at the onset of Kawasaki disease was 4.0±3.6 (median 3, range 0.2–14) years (*n*=64), and at the time of operation it was 13.3±11.1 (median 10, range 0.7–53) years (*n*=135) with an interval between the onset and the surgical operation of 9.5±9.4 years (median 6 years, range 10 days–41 years) (*n*=72).

The major presentations of this patient group are listed in Table 1. A total of 184 (28.9%) patients had at least once myocardial infarction, 33 of the 184 (17.9%) patients had 1–4 times of re-infarction. The locations of (re)infarction of 130 patients are listed in Table 2.

**Table 1:** Major presentations at the time of cardiac surgery

Major presentation	*n* (%)
Chest pain	16 (50)
Chest discomfort	4 (12.5)
Cardiac arrest	3 (9.4)
Chest pain + syncope	2 (6.3)
Shortness of breath	2 (6.3)
Syncope	1 (3.1)
Palpitation	1 (3.1)
Cyanosis	1 (3.1)
Fever, epigastric pain	1 (3.1)
Fever, malaise	1 (3.1)

**Table 2:** The locations of (re)infarction of 130 patients

(Re)infarction location	*n* (%)
Inferior	53 (40.8)
Anteroseptal	35 (26.9)
Lateral	19 (14.6)
Posterior	6 (4.6)
Non-Q wave	5 (3.8)
Anterior	3 (2.3)
Right ventricular	3 (2.3)
Anterolateral	2 (1.5)
Anteroinferior	1 (0.8)
Apical	1 (0.8)
Inferior, posterior	1 (0.8)
Anteroseptal, inferior	1 (0.8)

Eleven patients who did not have myocardial infarction had their electrocardiograms examined, which illustrated ST depression in five (45.5%) (one of them had ST depression in Master 2-step test, but his resting electrocardiogram was normal), ischemic changes in one (9.1%), subendocardial ischemia in two (18.2%), and normal in three (27.3%) patients, respectively. Echocardiography was a diagnostic tool in 33 patients: coronary artery aneurysm in 17 (51.5%), regional wall abnormality (hypokinesis, or akinesis) in four (12.1%), coronary artery dilation in two (6.1%), coronary artery calcification in two (6.1%), left ventricular aneurysm in one (3.0%), intra-coronary aneurysm thrombus in two (6.1%), left ventricular dysfunction+global hypokinesis+left ventricular aneurysm+coronary artery aneurysm in one (3.0%), and normal in three (9.1%) patients, respectively. Perfusion scintigraphy was used in at least three patients for the assessment of the left ventricular function of the patients (36, 48, 55).

The left anterior descending coronary artery (LAD), right coronary artery (RCA) and left main coronary artery (LM) were the most commonly involved coronary arteries in patients presenting with Kawasaki disease (Table 3). Stenosis, total occlusion and aneurysm were the three most common pathological changes of the coronary arteries. The locations of the pathological changes were reported in 39 patients: in the proximal coronary artery in 35 (89.7%), and in the mid, proximal-mid, proximal-distal, and distal segments of the coronary arteries in one (2.6%) patient each. The sizes of the reported coronary aneurysms were 20.2±15.6 mm (median 16, range 4.9–76.3), without significant difference between the sizes of the aneurysms of the LAD and RCA (18.0±10.5 mm vs. 22.5±19.9 mm, *p*=0.4600). The three LM aneurysms were 6.8, 13 and 20 mm, and the three aneurysms of the circumflex coronary artery were 6, 6 and 17 mm in diameter, respectively. In 58 (9.1%) patients, concurrent aneurysm and stenosis lesions developed in 86 coronary arteries including 46 (53.5%) LADs, 28 (32.6%) RCAs, 8 (9.3%) LMs, and 4 (4.7%) circumflex coronary arteries. Other findings included coronary artery ectasia in five (16.7%) (21, 47, 55, 56), thrombosed coronary aneurysms in nine (30%), calcified coronary aneurysms in 14 (46.7%), proximal RCA dissection in one (3.3%) (70), and left ventricular aneurysm in one (3.3%) patient (28), respectively.

**Table 3:** The pathological changes of the coronary arteries

Coronary artery	Stenosis, *n* (%)	Complete occlusion, *n* (%)	Aneurysm, *n* (%)	Total, *n* (%)
Left main coronary artery	9 (1.2)	2 (0.3)	101 (13.7)	112 (15.2)
Left anterior descending coronary artery	76 (10.3)	28 (3.8)	220 (29.8)	324 (43.9)
Diagonal branch	1 (0.1)			1 (0.1)
Ramus	1 (0.1)			1 (0.1)
Circumflex branch	5 (0.7)	3 (0.4)	57 (7.7)	65 (8.8)
Obtuse marginal branch	3 (0.4)		1 (0.1)	4 (0.5)
Right coronary artery	59 (8.0)	16 (2.2)	154 (20.8)	229 (31.0)
Posterior descending coronary artery	1 (0.1)			1 (0.1)
Posterolateral ventricular branch	1 (0.1)	1 (0.1)		2 (0.3)
Total	156 (21.1)	50 (6.8)	533 (72.1)	739 (100)

Four (0.6%) patients underwent heart transplantation, one (0.2%) patient had repair to an LM rupture (25), and 632 (99.2%) patients received CABG. A total of 628 (99.4%) patients received conventional CABG under standard cardiopulmonary bypass (one of them underwent a redo-off-pump coronary artery bypass (OPCAB) due to failure of the previous graft and the progression of the coronary pathology (50)), three (0.5%) patients underwent OPCAB (56, 65, 72), and one (0.2%) patient received minimally invasive direct coronary artery bypass (62). There were 1,425 grafts with 1,445 distal anastomoses in CABGs including 1,409 independent grafts with 1,409 anastomoses, nine sequential grafts with 22 anastomoses, three composite Y-grafts with nine anastomoses, and four composite I-grafts with five anastomoses, respectively (Table 4). A total of 388 patients had their graft number recorded: 193 (49.7%) patients had one graft, 144 (37.1%) patients had two grafts, 39 (10.1%) patients had three grafts, 10 (2.6%) patients had four grafts (including one patient receiving a redo-OPCAB (50)) and two (0.5%) patients had five grafts with a mean of 1.67±0.80 grafts/patient. There were 819 receipt coronary arteries and 782 graft vessels that were recorded in detail (Table 5). Left internal mammary artery (IMA) to LAD was the most commonly used bypass graft received by 170 (26.7%) patients. The secondary procedures to CABG were LM aneurysm repair in seven (28%), and hybrid stenting of the proximal obtuse marginal branch in one (4%) (20), Ramus stenting in one (4%) (38), intracoronary aneurysmal thrombus extraction in two (8%), RCA plication/aneurysmorrhaphy in nine (36%), LAD plication in two (8%), RCA aneurysm ligation in one (4%), LAD ligation in one (4%), left coronary artery plication in one (4%) patient, respectively. Graft patency was evaluated in 187 patients for 21.4±32.3 (range 0.1–252) months: 154 (82.4%) grafts (both the arterial and the saphenous) were patent and 33 (17.6%) were occluded and some of them were treated by stenting or redo-CABG. No significant difference was found between the term of patency of the IMA and that of other arterial grafts (the radial and right gastroepiploic arteries) (22.3±33.6 months vs. 7.8±7.6 months, *p*=0.8006), or between that of the arterial grafts and of the saphenous vein grafts (SVGs) (21.5±32.8 months vs. 19.6±26.1 months, *p*=0.7848), even though the duration of the IMA tended to be longer than that of the other arterial grafts and that of the arterial was longer than that of the SVGs.

**Table 4:** Summary of the grafts and anastomoses in Kawasaki disease patients requiring coronary artery bypass grafting

Graft configuration	Grafts, *n* (%)	Distal anastomoses, *n* (%)
Independent	1,409 (98.9)	1,409 (97.5)
Sequential	9 (0.6)	22 (1.5)
Composite Y-graft	3 (0.2)	9 (0.6)
Composite I-graft	4 (0.3)	5 (0.4)
Total	1,425 (100)	1,445 (100)

**Table 5:** Receipt and donor vessels of coronary artery bypass grafting

Receipt/donor vessel	*n* (%)
Receipt coronary vessel	819
Left anterior descending coronary artery	450 (54.9)
Right coronary artery	214 (26.1)
Circumflex branch	88 (10.7)
Diagonal branch	28 (3.4)
Obtuse marginal branch	14 (1.7)
Posterior descending coronary artery	14 (1.7)
Posterior left ventricular branch	6 (0.7)
Left coronary artery	3 (0.4)
Acute marginal branch	1 (0.1)
Atrioventricular branch	1 (0.1)
Donor vessel	782
Internal mammary artery (either the left or the right, but not clearly indicated in the literature)	322 (41.2)
Left internal mammary artery	173 (22.1)
Saphenous vein	127 (16.2)
Right gastroepiploic artery	62 (7.9)
Right internal mammary artery	57 (7.3)
Radial artery	40 (5.1)
Inferior epigastric artery	1 (0.1)

There were four early deaths and 19 late deaths at 3 months–16 years after the operation with an early and a late mortality of 0.6 and 3.0%, respectively.

## Discussion

The coronary artery stenotic lesions in Kawasaki disease commonly involve severe calcification, whereas adult coronary artery lesions prevail with atherosclerosis (79). Severe calcified coronary artery, the patient's small body weight and the lack of suitable sized burr were the risk factors leading to failed stent implantation in pediatrics, and therefore CABG could be an alternative (80).

Coronary arteries may be occluded suddenly in Kawasaki disease with thrombus formation in the aneurysm (34). Thrombus can easily develop in the coronary aneurysms despite strict anticoagulant therapy for the patients with Kawasaki disease (17). Acute myocardial infarction occurred with most frequency within 2 years of the onset of Kawasaki disease and was mainly caused by fresh thrombus (81). Occlusion of the RCA may induce not only inferior infarction of the left ventricle but also right ventricular infarction and fatal heart block, and hence a sole RCA occlusion is still an indication of coronary artery revascularization (82). Remarkably, occlusion of the LM was sometimes a congenital disorder but not a sequela of Kawasaki disease (75).

An inflammatory infiltrate in the lymphocytes, macrophages and immunoglobulin A plasma cells in the coronary arteries constitute the pathogenesis of the coronary pathologies (17). Histologically, coronary arteritis showed edematous dissociation of the tunica media 6–8 days after the onset of Kawasaki disease. On the tenth day of the disease, the lymphocytes and macrophages began to infiltrate the arterial wall, spreading into all layers of the artery, resulting in arterial structures being severely damaged. Aneurysms developed on about the 12th day after the onset when the damage was severe. The blood eddied in the aneurysm, and thrombi easily formed (5). Immunoglobulin A plasma cells may infiltrate vasculitis lesions with many monocytes/macrophages and CD8 T lymphocytes (83). Intimal calcification of the coronary arteries may hinder the normal growth of the arteries and result in stenotic lesions (33).

Intravenous gamma globulin and appropriate doses of aspirin such as 3 mg/kg/day are ways of treatment at the acute stage (19). Treatment with corticosteroids, immunoglobulines and acetylicsalicylic acid may reduce the incidence of cardiac complications in Kawasaki disease. Percutaneous transluminal coronary angioplasty was indicated for localized severe stenotic lesions (≥75%) not involving the ostia, but showed a lower efficacy in comparison with CABG (81). CABG should be recommended in younger children who have ischemic changes with multivessel disease (69).

The IMA graft in pediatric patients with Kawasaki disease can increase in length and diameter because the IMA is a living graft and, therefore, has a self-regulating function (84). Because the child's SVG is too small, an SVG harvested from the mother can be an alternative for bypassing the RCA with good patency (85). Clinical observations revealed patency rates of the arterial grafts to be 94, 82 and 78% at 1, 5 and 10 years, respectively, and this was higher than that of the respective venous grafts (82, 63 and 36%) (86). The 10-, 20-, and 30-year survival rates after the onset of Kawasaki disease were 95, 88, and 88%, respectively (78).

Hsu et al. (56) adopted OPCAB technique for multiple arterial grafts in an adult patient with Kawasaki disease. Kowalczyk et al. (65) performed bilateral IMA grafts with OPCAB in a 3-year-old-boy with Kawasaki disease. Verma et al. (72) grafted the right IMA to distal RCA with OPCAB in a 6-year-old girl. Takata et al. (62) performed composite arterial grafts by robotically assisted, minimally invasive, direct coronary artery bypass for a patient with Kawasaki disease. All of the above coronary lesions due to Kawasaki disease were treated successfully with either OPCAB or minimally invasive direct coronary artery bypass, but long-term follow-up was warranted.

In general, patients with Kawasaki disease were very young, some requiring a cardiac surgical procedure at a very early age. The interval between the onset and the surgical operation was 9.5±9.4 years. The prevalence of myocardial infarction and re-infarction was high. LAD, RCA and LM were the most commonly involved coronary arteries with 89.5% of the lesions located in the proximal coronary artery. Giant aneurysm, critical stenosis with calcification and thrombus formation of the coronary arteries often led to a CABG, heart transplantation or coronary arterial aneurysm plication. Left IMA to LAD was the most commonly used bypass graft of CABG. Graft patency rate was 82.4% at 21.4±32.3 (range 0.1–252) month follow-up. The early and late mortalities of this patient setting were 0.6 and 3.0%, respectively.

In conclusion, patients with Kawasaki disease may develop coronary artery lesions and warrant CABG at a very early age. With left IMA to LAD anastomosis as the first choice of bypass graft, the long-term patency and patient survival were satisfactory.
